# iReport: a generalised Galaxy solution for integrated experimental reporting

**DOI:** 10.1186/2047-217X-3-19

**Published:** 2014-10-13

**Authors:** Saskia Hiltemann, Youri Hoogstrate, Peter van der Spek, Guido Jenster, Andrew Stubbs

**Affiliations:** 1 Department of Bioinformatics, Erasmus Medical Center, Wytemaweg 80, 3015 CN Rotterdam, The Netherlands; 2 Department of Urology, Erasmus Medical Center, Wytemaweg 80, 3015 CN Rotterdam, The Netherlands

**Keywords:** Galaxy, Generic reporting application, Next generation sequencing, Genetic variation, Pathogenic gene selection

## Abstract

**Background:**

Galaxy offers a number of visualisation options with components, such as Trackster, Circster and Galaxy Charts, but currently lacks the ability to easily combine outputs from different tools into a single view or report. A number of tools produce HTML reports as output in order to combine the various output files from a single tool; however, this requires programming and knowledge of HTML, and the reports must be custom-made for each new tool.

**Findings:**

We have developed a generic and flexible reporting tool for Galaxy, iReport, that allows users to create interactive HTML reports directly from the Galaxy UI, with the ability to combine an arbitrary number of outputs from any number of different tools. Content can be organised into different tabs, and interactivity can be added to components. To demonstrate the capability of iReport we provide two publically available examples, the first is an iReport explaining about iReports, created for, and using content from the recent Galaxy Community Conference 2014. The second is a genetic report based on a trio analysis to determine candidate pathogenic variants which uses our previously developed Galaxy toolset for whole-genome NGS analysis, CGtag. These reports may be adapted for outputs from any sequencing platform and any results, such as *omics* data, non-high throughput results and clinical variables.

**Conclusions:**

iReport provides a secure, collaborative, and flexible web-based reporting system that is compatible with Galaxy (and non-Galaxy) generated content. We demonstrate its value with a real-life example of reporting genetic trio-analysis.

## Findings

Structured reporting and documentation of experimental outcome is required for the successful transfer of knowledge from the research scientist to their peers and to the broader academic community.

Galaxy is a platform that aims to provide complex bioinformatics services and tools in an easy-to-use web-based graphical user interface [1-3]. The output from these tools can be displayed using built-in Galaxy visualisation applications [4], via specialised visuals implemented as a component in the workflow deployed in Galaxy [5] or by downloading the results and visualising the output with applications external to Galaxy (e.g., Excel, TIBCO spotfire, R, spreadsheet programs, *etc*).

Galaxy has the capacity to track the provenance of the source data, the workflow, as well as the workflow components used to analyse the data. Currently users can share their workflow and results within Galaxy, but do not have access to a simple method to summarise results from multiple tools and/or workflows in an integrated report. To address this issue we have developed iReport, an integrated reporting application that provides users with a flexible means to produce dynamic HTML reports which can be shared with other Galaxy users or downloaded to disk.

Systems used by end-users to deliver graphical output range from open-source applications, such as *Ad Hoc* reports [6], Google charts (and docs) [7] and OpenOffice [8], to commercial applications such as Microsoft Office. Indeed scientific reporting applications both open-source (Bioconductor [9], Circos [10,11]) and commercial software (e.g., Omniviz [12], Partek [13]) include a multitude of visualisation capabilities with a focus on data reporting and presentation of data in the context of the experimental design and with associated meta-data. There are some applications, like TIBCO spotfire [14], which are capable of integrating results from multiple sources including associated text and meta-data and other applications which serve as an electronic lab note book (e.g., IDBS [15]). Additionally there have been many products developed to address the selection and reporting of variants for pathogenic variant selection including the workflow to identify those variants (e.g., Gensight [16], Cartagenia [17], Clinical Genomicist [18]). For data generated in R, dynamic reporting packages such as KnitR [19], Sweave [20] and R-Markdown [21], allow for the integration of data-generating code within the report specification itself. Similar systems exist for other programming languages, for example Tangle [22] (JavaScript), Active Markdown [23] (CoffeeScript) or IPython Notebooks [24] (Python). These are very versatile tools, but require programming knowledge to use effectively. iReport offers an open-source application for both Galaxy and non-Galaxy produced results allowing for the generation of customized integrated reports for any type of project or workflow. The advantage to Galaxy users is that the output from any application can be included into any report, and that a report template can be reused for other projects. Also, the report may be securely shared with one or many users of that Galaxy instance or made publicly available. iReports can be completely configured from the tool web interface, and requires no programming or knowledge of the underlying system.

We demonstrate iReport’s utility through an example where a genetic report is generated from outputs of an existing Galaxy next-generation sequencing (NGS) toolkit, CGtag [5]. iReport can also be used as an electronic lab notebook by creating an iReport which links out to various other iReports containing different analysis reports from various samples. It can be also be coupled to output from other Galaxy instances, for example output generated by specialized Galaxy instances such as Confero [25], ORIONE [26], and Galaxy-P [27].

### Functionality

iReport dynamically generates HTML, and employs JavaScript and jQuery to create interactive components, such as searchable, sortable, paginated tables and zoomable images. iReport is ideally suited to use as the final step in a workflow; the pipeline developer configures the report once and end-users are then presented with a templated report each time users run the workflow, while only needing to provide the input files for the pipeline [28]. iReport can also be used directly by end-users as a means of easily sharing their results with other Galaxy users, or the public via Galaxy’s native sharing capabilities.

The generic reporting functionality and usage of iReport is outlined below using an example iReport created for the recent Galaxy Community Conference, which is also available for viewing online [29]. It is followed by an example of a genetic report that can be used for trio analysis, which can easily be modified for any trio reporting or extended to quartets or larger families, also available from our demo galaxy [30].

#### iReport structure

iReport produced a report webpage consisting of one or more subpages with one or multiple elements included on each subpage. The primary output of iReport is: 

1. A cover page 

(a) Title of the report

(b) Cover image

2. Main report page consisting of a set of tabs. Each tab consisting of one or more *content items*. Each content item can be one of the following types: 

(a) Text

(b) Images

(c) Tables

(d) PDF Files

(e) Links

An iReport tutorial has been developed to demonstrate and explain the functionality of iReport, and is available as a shared history from the CTMM-TraIT public Galaxy instance [29]. The following sections describe each of the components of iReport in more detail.

#### Cover page

**Figure 1:**
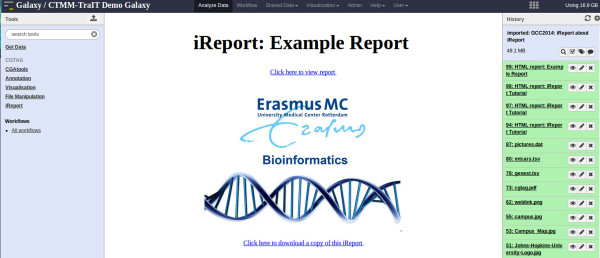
**Example cover page.** Example of a cover page with title *Example Report* and the default cover image. A link to download the entire iReport web page is also provided.

The cover page consists of a user-specified title and a cover image. The cover image parameter is optional and when the field is left blank a default image is used (Figure 1). By clicking on the image, or the link above it, the user can access the main report page. There is also a link to download the entire iReport webpage, including all dependency files, for storing or viewing on different systems.

#### Main report page

**Figure 2:**
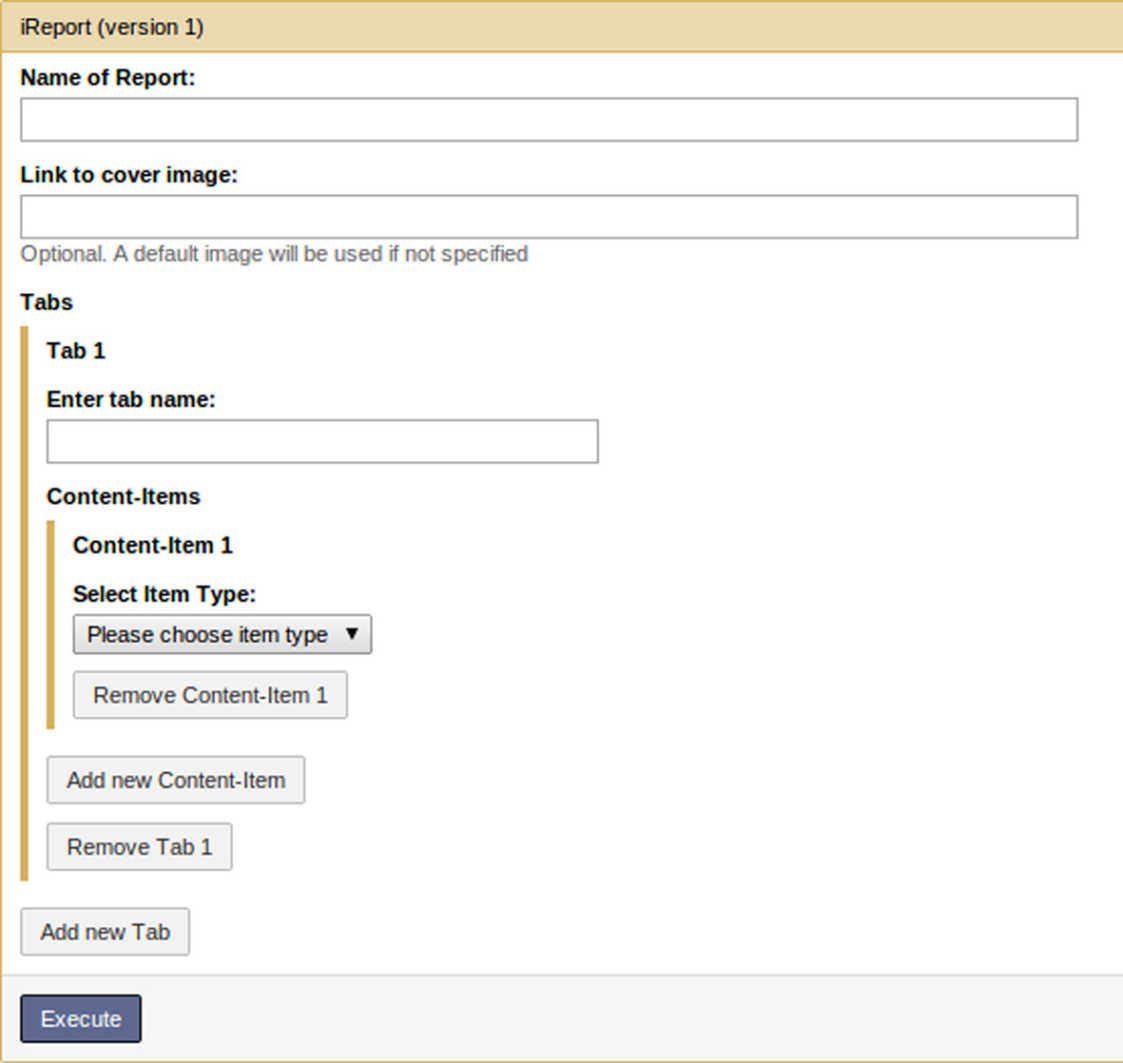
**iReport tool wrapper.** iReport’s tool interface. Minimally a report title and at least 1 tab with 1 content item need to be specified.

An arbitrary number of tabs may be added via a repeat parameter. Each tab can be labelled with a name specified by the user. An arbitrary number of *content items* may then be added to each tab in a repeat parameter. A type must be specified for each content item (e.g., text, image, table *etc.*), as well as several other parameters depending on the type chosen (Figure 2). Layout is mostly left up to the browser, but users can explicitly add a line-break after each item to force items to appear underneath each other.

##### Content item: text field

Text can be entered in a text field in the tool interface, for example to create an introduction paragraph and to give a description of the items on the page. Text is printed verbatim, although a small number of HTML tags are allowed in order to give the user some control over formatting (e.g., b,i,em, strong, h1-h6 tags). Text files can also be specified, and the contents of the file will be printed to the screen verbatim.

##### Content item: images

**Figure 3:**
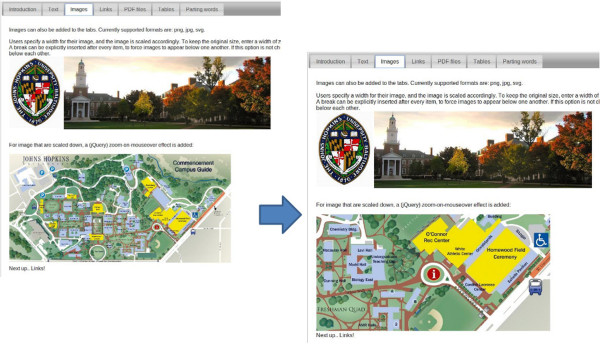
**Zoom effect.** Images that have been scaled down can optionally be enhanced with a jQuery zoom-on-mouseover effect. In this example, the bottom image has this effect added, and when the user moves their mouse over the image, a zoomed version of that area of the image is shown.

Many tools produce images as output, which can also be displayed by iReport. Users specify the image file from their Galaxy history, and the desired image size. For images that have been scaled down, an optional jQuery zoom-on-mouseover effect may be added (Figure 3) [31]. Currently supported image formats are JPG, PNG and SVG.

##### Content item: tables

**Figure 4:**
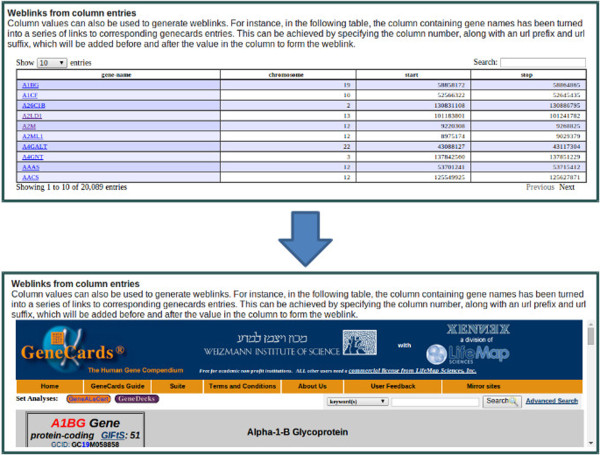
**Weblinks from table columns.** A series of web links can be created within a table by specifying a prefix and suffix to be placed before and after each column entry.

iReport can also display tables. The input must be a tab-delimited file from the users’ Galaxy history, and the first nonempty line not starting with a hash symbol (#) is assumed to contain the column headers. The jQuery library *DataTables*[32] is used to create tables which can be searchable, sortable and paginated, if requested by the user. There is an option to create hyperlinks within the columns of a table by providing a column number, a URL prefix and a URL suffix. This is illustrated in Figure 4, where the first column contains gene names and by including the GeneCards [33,34] URL prefix “http://www.genecards.org/cgi-bin/carddisp.pl?gene=”. This generates a hyperlink to the corresponding GeneCards entry for every item in the column in the table.

##### Content item: PDF files

**Figure 5:**
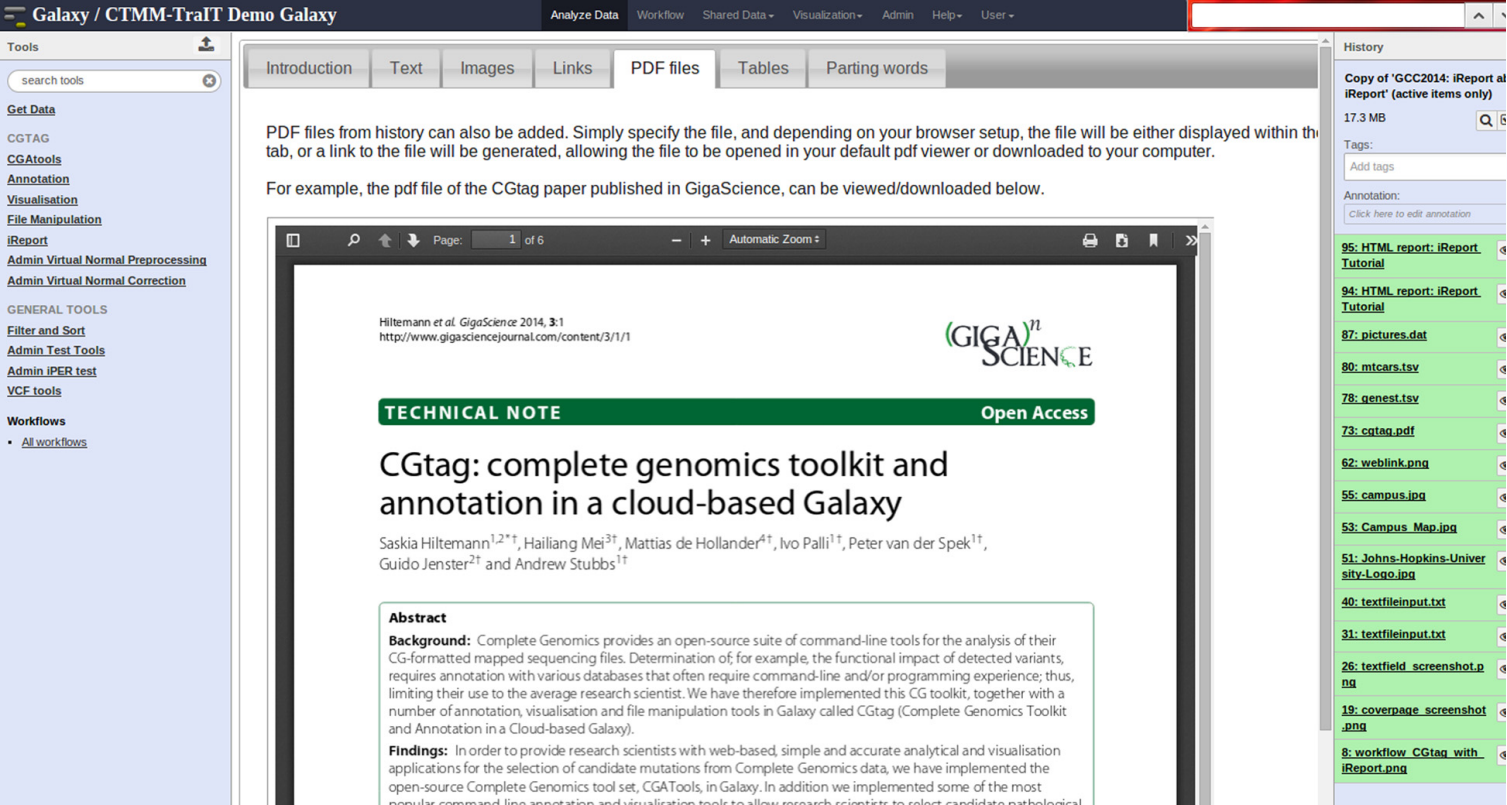
**Embedded PDF files.** iReports can also display PDF files. For browsers without PDF plug-in, a download link to the file will be created instead.

This is one of the simplest content items. The user provides a PDF file from the Galaxy history, which will be embedded in the page. If the browser does not have the necessary plug-ins installed, a download link for the file will be generated instead (Figure 5).

##### Content item: links

**Figure 6:**
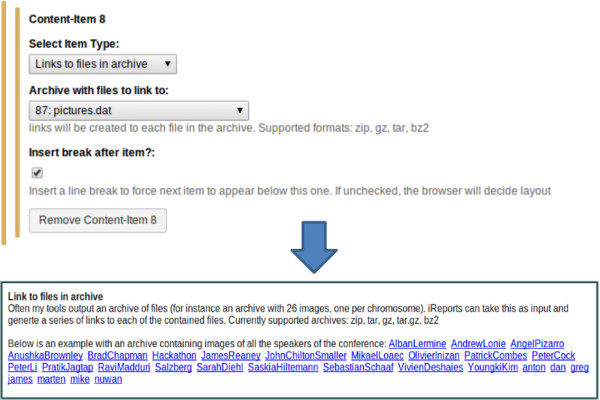
**Links to all files in an archive.** Given an archive of files, iReport can create a series of links to all files contained in the archive. Link texts are the filenames (without file extension).

Users can create links to web locations by specifying a URL and a link text. Links to datasets in the history can also be created here by specifying a dataset and a link text. Several tools create archives of files as output (for example a zip file containing the plots for each chromosome). Links to all files contained in an archive can also be created, and will be named with the file names (excluding file extension). Currently the supported archive formats are zip, bz2, tar, gz and tar.gz. An example can be seen in Figure 6, where an archive with images was used as input and a series of links to each contained file was created. An option to create a link to an iReport is also present. This allows users to create a kind of electronic lab notebook, by creating an overview of all their samples and linking to one or more iReports for each sample.

### Genetic report for a trio of HapMap individuals

Accurate, reproducible and traceable reporting is an essentialrequirement to the evaluation of the genetic outcome from any assay [35], including those variations predicted from NGS analysis. Since iReport is capable of including many formats, we have used the outcome from a trio analysis generated from the Complete Genomics [36] NGS platform to demonstrate its utility in representing these data in a user-defined format, which contains the provenance of the underlying analysis. In this example we use a trio of individuals sequenced in the International HapMap Project [37,38], to demonstrate how to select protein affecting candidate variants based on a recessive genetic model. All data in this example is freely available for download from the Complete Genomics website [39].

**Figure 7:**
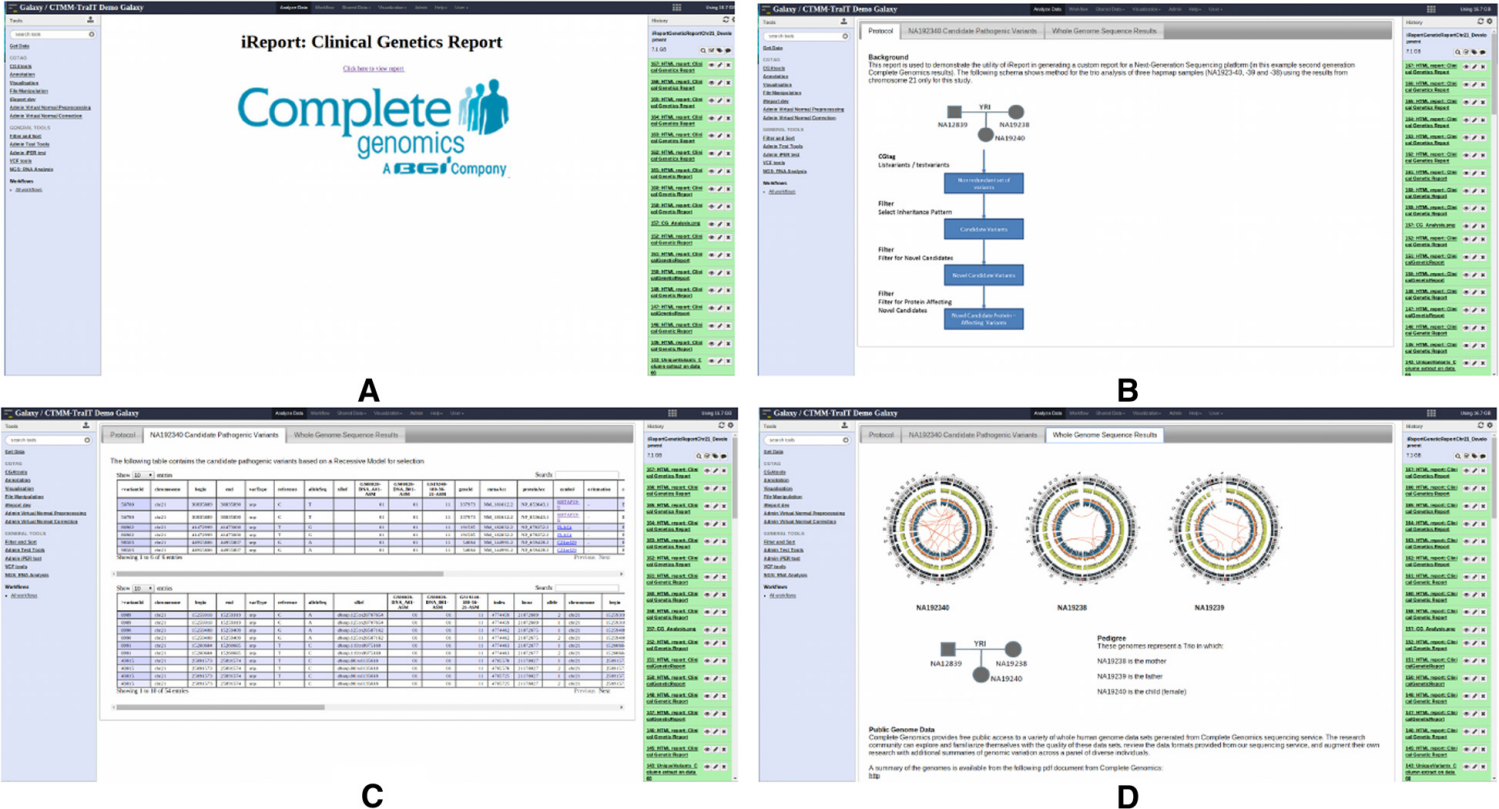
**Example iReport: Genetic Report.** Example iReport for Clinical Genetics. **A)** Cover page with custom image. **B)** First tab, explaining the protocol used. **C)** Second tab, tables of candidate pathogenic variants, gene columns linking out to GeneCards. **D)** Fourth tab showing Circos images and family structure.

This example iReport has one tab devoted to explaining the protocol used (Figure 7B), one tab with circos plots and an explanation of the family structure (Figure 7D), and one tab with tables containing the candidate pathogenic variants determined by the protocol based on a recessive model for selection. This iReport is also available as a published history on the TraIT-CTMM public Galaxy [40].

### Conclusions

iReport is a easy-to-use, flexible tool for generating traceable, standardized reports which are easily shared between users within and across platforms. We have demonstrated that iReport is capable of creating a customised genetics report from results generated within Galaxy and may be shared with collaborators on the same platform, or with the public. Additionally, data or results generated externally can be uploaded into Galaxy and can also be used by iReport. These reports are generated as web pages and may be downloaded in their entirety to be easily shared across systems.

The genetics report presented here represents the bare minimal reporting that is required to summarise the output for a genetic variation analysis. Whilst we used a trio of individuals to demonstrate how to select protein-affecting candidate variants based on a recessive model, any number of model outcomes and other assay results may be included in an iReport.

We developed iReport to simplify reporting and sharing the output from *omics* and non-high throughput assays analysed both in and external to Galaxy. We have also utilised iReport for more complex analysis workflows, such as summarising translational research and diagnostic applications for cancer and immunological research and diagnostics.

## Availability and requirements

**Project name:** iReport **Project home page:**https://github.com/shiltemann/iReport**CTMM-TraIT public Galaxy instance:**http://galaxy.ctmm-trait.nl**iReport tool shed repository:**https://toolshed.g2.bx.psu.edu/view/saskia-hiltemann/ireport**Operating system(s):** Unix-based Operating Systems **Programming languages:** Bash, Perl, Python **Other Requirements:** Galaxy **License:** GNU GPL **Any restrictions to use bynon-academics:** none **Examples:iReport about iReport published history:**http://galaxy.ctmm-trait.nl/u/saskia-hiltemann/h/gcc2014-ireport-about-ireport,ortinyurl.com/llrzz9w**Clinical Genetics iReport published history:**http://galaxy.ctmm-trait.nl/u/andrew-stubbs/h/ireportgeneticreportchr21

## Availability and supporting data

The iReport tool, user manual (published page), and example data and histories are available at the CTMM-TraIT Galaxy server [40].

## Abbreviations

CGtag: Complete genomics toolkit and annotation in a cloud-based galaxy; CTMM-TraIT: Center for Translational Molecular Medicine - Translational IT; NGS: Next generation sequencing; URL: Uniform resource locator.

## Competing interests

The authors declare that they have no competing interests.

## Authors’ contributions

SH, GJ, and AS contributed to the design and coordination of iReport and manuscript preparation. SH and AS contributed to implementing iReport. SH, GJ, YH, PvdS and AS contributed to testing of iReport and all authors read and approved the final manuscript.
